# Does Clinical Training Influence Empathy in Dental Students? Evidence from a Cross-Sectional Study in Lithuania

**DOI:** 10.3390/dj14030137

**Published:** 2026-03-02

**Authors:** Kornelija Rogalnikovaitė, Julija Narbutaitė, Vilija Andruškevičienė, Vilma Brukienė, Eglė Aida Bendoraitienė

**Affiliations:** 1 Department of Preventive and Pediatric Dentistry, Faculty of Odontology, Medical Academy, Lithuanian University of Health Sciences, J. Lukšos-Daumanto Str. 6, 50106 Kaunas, Lithuania; julija.narbutaite@lsmu.lt (J.N.); vilija.andruskeviciene@lsmu.lt (V.A.); egleaida.bendoraitiene@lsmu.lt (E.A.B.); 2 Institute of Dentistry, Faculty of Medicine, Vilnius University, Žalgirio Str. 115, 08217 Vilnius, Lithuania; vilma.brukiene@mf.vu.lt

**Keywords:** empathy, dental students, clinical training, patient-centered care

## Abstract

**Background/Objectives**: Empathy is a core component of professional competence in dentistry, influencing patient-centered care and treatment outcomes. Evidence suggests that empathy may decline during clinical training, but data from Lithuanian dental students are lacking. This study aimed to assess empathy levels and subscale patterns among Lithuanian dental students and examine their association with academic year. **Methods**: A cross-sectional study was conducted among third- to fifth-year dental students at the two universities in Lithuania. The Lithuanian version of the Jefferson Scale of Empathy–Health Professions Students (JSE-HPS) was used to measure total empathy and three subscales: Perspective Taking (PT), Compassionate Care (CC), and Standing in the Patient’s Shoes (SPS). Internal consistency was assessed using Cronbach’s alpha. Factor validity was examined via principal component analysis with Varimax rotation and Kaiser normalization. Differences across academic years were analyzed using Kruskal–Wallis tests. **Results**: A total of 252 students completed the questionnaire (response rate: 93%). The Lithuanian JSE-HPS demonstrated good internal consistency (α = 0.808) and confirmed a three-factor structure. The mean total empathy score was 106.07 ± 12.55. JSE-HPS scores differed significantly between dental classes (*p* < 0.001). Fifth-year students had significantly lower JSE-HPS scores than third- and fourth-year students (101.65 vs. 107.05 and 109.36; *p* = 0.035 and *p* = 0.007). PT and CC scores significantly declined in fifth-year students compared with earlier years, whereas SPS scores remained stable. **Conclusions**: The Lithuanian version of the JSE-HPS is a reliable and psychometrically sound tool for assessing empathy. Clinical training was significantly associated with a decline in total empathy scores among Lithuanian dental students, highlighting the impact of academic progression on both cognitive and affective components of empathy. Given the cross-sectional design, causal inferences cannot be drawn.

## 1. Introduction

The primary goal of dental education is to equip students with the knowledge and skills necessary to practice dentistry competently [1]. Professionalism, a critical component of competent practice, is a multifaceted construct encompassing behaviors and attitudes that extend beyond technical expertise [2]. In Europe, the Association for Dental Education has outlined competencies emphasizing ethical conduct, adherence to professional codes, and social skills, with particular attention to effective communication and patient-centered care [3].

Empathy, a fundamental element frequently incorporated into various definitions of patient-centered care [4,5], may influence a healthcare professional’s patient-centered approach. It is defined as ‘the ability to understand the patient’s situation, perspective, and feelings, and to communicate that understanding to the patient’ [6]. Empathy is recognized as a core component of professional competence and is central to establishing and maintaining effective clinician–patient relationships [7,8]. In general, empathetic behavior has been positively associated with reduced pain, anxiety, depression, and distress, as well as with improved self-management, patient satisfaction, and sense of enablement [9,10,11]. In dentistry, empathy is particularly important as it fosters trust, reduces patient anxiety and fear, and enhances communication between dentist and patient. By integrating empathy into clinical interactions, dentists can improve patients’ emotional comfort, strengthen the therapeutic alliance, and contribute to better adherence, satisfaction, and overall treatment outcomes [12].

Promoting empathy among students is a key objective of clinical dental education [13]. However, despite its acknowledged importance in dental training and patient-centered care, empirical evidence suggests that empathy may decline as students transition from preclinical to clinical stages of dental education. In 2005, Sherman and Cramer reported that first-year dental students demonstrated significantly higher empathy scores compared with students in all subsequent years of dental school [14]. More recent studies continue to describe a trend toward declining empathy; however, overall findings remain inconclusive. In France, empathy scores were found to decrease gradually, though not significantly, during clinical training [15]. A systematic review by Narang et al. [16] further suggested that increased patient exposure is frequently associated with reduced empathy among dental students. Conversely, studies by Nazir et al. [17] and Torres-Martínez et al. [18] reported that empathy levels tended to increase as students advanced to higher academic years.

Despite growing international interest in empathy development during dental education, evidence from Lithuania is currently lacking. To date, no published studies have systematically examined empathy levels among Lithuanian dental students or explored how clinical training and patient exposure may influence empathy across different stages of dental education. Given the inconclusive findings reported in other countries and the potential influence of cultural, educational, and healthcare system differences on professional attitudes, data from Lithuania are needed to better understand national trends. Therefore, this cross-sectional study aims to assess empathy levels among dental students in Lithuania and to investigate the association between clinical practice and empathy development.

## 2. Materials and Methods

### 2.1. Ethical Approval

This study was conducted in accordance with the Declaration of Helsinki and was approved by the Bioethics Centre of the Lithuanian University of Health Sciences (No. 2025-BEC2-0085, 16 January 2025).

### 2.2. Study Population

This cross-sectional study was conducted among dental students enrolled in Lithuanian dental education programs during the 2024–2025 academic year at the Lithuanian University of Health Sciences (LSMU) and Vilnius University (VU), the two principal institutions providing dental training in Lithuania. The study population comprised students in their third to fifth years of study, as these cohorts had commenced clinical training and were engaged in direct patient care. During the study period, 57 students were enrolled at VU and 213 at LSMU.

A total of 270 paper surveys were distributed to eligible dental students immediately prior to scheduled lectures or mandatory practical sessions during the spring term of the academic year. Before questionnaire administration, the aim of the study was clearly explained to all participants. Participation was voluntary and anonymous, and students were informed of their right to withdraw at any stage without consequence. Completion and return of the questionnaire were regarded as implied informed consent to participate in the study. To maintain anonymity, completed questionnaires were returned by participants into a collection box placed out of the distributors’ view.

### 2.3. Questionnaire

The students’ empathy levels were measured using the Lithuanian version of the Jefferson Scale of Empathy–Health Professions Students (JSE–HPS). Permission to use the JSE–HPS was obtained from the Center for Research in Medical Education and Health Care, Jefferson Medical College, Thomas Jefferson University, USA.

In the first stage of the validation process, two translators (K.R. and E.B.) performed a forward translation. To verify the validity of this version, two other bilingual researchers (J.N. and V.A.) conducted a blinded back-translation into the original language. The preliminary questionnaire was then pilot-tested with 25 dental hygiene students to assess the clarity, comprehensibility, and potential bias of each item. Participants were asked to provide feedback on item wording, ambiguity, ease of understanding, and whether any items could be interpreted in a socially desirable or leading manner. Items identified as unclear, redundant, or potentially bias-inducing were revised accordingly. Item–total correlations were also examined to detect inconsistent response patterns. The Cronbach’s alpha coefficient for the final version was 0.838, indicating good internal consistency and reliability. Based on these procedures, the final Lithuanian version of the JSE–HPS was approved for use.

The JSE–HPS is a 20-item scale. Each item is rated on a 7-point Likert scale ranging from 1 (strongly disagree) to 7 (strongly agree). The scale evaluates three factors: (a) perspective taking (PT; 10 items: 2, 4, 5, 9, 10, 13, 15, 16, 17, and 20); (b) compassionate care (CC; 8 items: 1, 7, 8, 11, 12, 14, 18, and 19); and (c) standing in the patient’s shoes (SPS; 2 items: 3 and 6). Ten of the twenty items are negatively worded and therefore reverse-scored. The total score, obtained by summing all item scores, ranges from 20 to 140, with higher scores indicating a greater level of empathy. No cut-off points were established.

### 2.4. Statistical Analysis

Statistical analyses were performed using SPSS software (version 30.0; IBM Corp., Chicago, IL, USA). Categorical variables were presented as frequencies and percentages. Continuous variables were summarized using the mean and standard deviation (SD), as well as the median with minimum and maximum values. The standard error of the mean (SE) was reported to indicate the precision of the mean estimates. The internal consistency of the JSE–HPS was assessed using Cronbach’s alpha (α). To examine the underlying factor structure of the JSE–HPS and assess interrelationships among its items, exploratory factor analysis (EFA) was performed. The suitability of the data for factor analysis was assessed using the Kaiser–Meyer–Olkin (KMO) measure of sampling adequacy and Bartlett’s test of sphericity, with a KMO value ≥ 0.50 and a significance level of *p* < 0.001 considered indicative of adequacy for EFA. Differences in empathy scores among the three student groups were analyzed using the Kruskal–Wallis test, followed by post hoc pairwise comparisons with Dunn’s test and Bonferroni correction for multiple testing. Pearson’s correlation coefficient (r) was used to assess pairwise associations between the dimensions of the JSE–HPS. Statistical significance was set at *p* < 0.05.

## 3. Results

### 3.1. Participants’ Flow and Characteristics

A total of 252 completed questionnaires were returned, yielding a response rate of 93%. The sample was predominantly female (88.89%), and most participants were enrolled at LSMU (78.57%). The approximate mean age was 24.04 ± 3.08 years, calculated using age-category midpoints. Participants were evenly distributed across academic years. Descriptive characteristics of the study sample are presented in Table 1.

### 3.2. Internal Consistency of the JSE–HPS

The total JSE-HPS demonstrated good internal consistency (Cronbach’s α = 0.808). Corrected item–total correlations exceeded the acceptable threshold of 0.30 for all items except four (#3, #6, #18, #19). Among the subscales, PT showed good reliability (α = 0.811), whereas CC and SPS exhibited questionable reliability (α = 0.639 and 0.630, respectively). The lower Cronbach’s alpha values observed for the CC and SPS subscales may be partly attributable to the small number of items, as internal consistency estimates are known to be sensitive to scale length.

### 3.3. Factorial Validity of the JSE-HPS

EFA of the Lithuanian version of the JSE–HPS was conducted using principal component extraction with Varimax rotation and Kaiser normalization. The data met the assumptions for factor analysis (KMO = 0.836; Bartlett’s test of sphericity, *p* < 0.001). Three factors were retained—PT, CC and SPS—with eigenvalues of 5.29, 1.62, and 1.40, explaining 41.53% of the total variance.

The rotated solution revealed a largely coherent factor structure. Most items loaded strongly on their theoretically expected factors. Item #8 showed a complex pattern: it did not reach the 0.40 threshold on CC (loading = 0.294) and displayed a marginally higher cross-loading on PT (loading = 0.383), suggesting closer alignment with PT in this sample; however, it was retained due to conceptual interpretability.

The CC factor exhibited loadings between 0.50 and 0.77. Items #10 and #16 showed moderate secondary loadings on PT alongside their primary CC loadings, reflecting overlap between cognitive and affective components of empathy. The SPS factor included three items with loadings from 0.45 to 0.80. Notably, item #18, inconsistently associated with SPS in prior studies, demonstrated its strongest loading on SPS, supporting its inclusion in this dimension. The observed item-level variations reflect expected measurement differences when the JSE-HPS is applied in different linguistic and educational contexts and do not undermine its overall construct validity. Table 2 shows the factor structure and item statistics.

### 3.4. Empathy Scores

The mean total empathy score was 106.07 (SD = 12.55), with a range of 67–135. Descriptive statistics by year of study are presented in Table 3. The Kruskal–Wallis test revealed a significant difference in JSE-HPS scores across third-, fourth-, and fifth-year students (*p* < 0.001). Post hoc analyses indicated that fifth-year students had significantly lower empathy scores compared with third- and fourth-year students (Table 4).

Descriptive statistics for subscale scores by academic year are presented in Table 5. Significant differences were observed for the PT and CC subscales (Kruskal–Wallis, *p* < 0.05), whereas SPS scores did not differ significantly between years. Post hoc comparisons demonstrated significantly lower PT scores among fifth-year students relative to third- and fourth-year students, while CC scores differed significantly between fourth- and fifth-year students (Table 6).

### 3.5. Inter-Correlations Between JSE-HPS Factors

Correlations between the JSE-HPS total and subscale scores are presented in Table 7. Total JSE-HPS scores were positively and significantly correlated with all three subscales. However, the SPS subscale did not show significant correlations with the PT or CC subscales.

## 4. Discussion

Professional development in dental education involves the gradual integration of clinical competence, professional identity, and interpersonal attitudes [19]. Empathy, as a fundamental professional attribute, is not a static trait but may fluctuate in response to educational demands and clinical exposure. The present findings indicate that this developmental trajectory in dental education may be characterized by a decline in empathy during the later years of training. The transition from preclinical to clinical training entails a variety of challenges that may influence students’ emotional and interpersonal engagement. While this shift marks a period of substantial personal and professional development, it is frequently associated with increased stress and anxiety [20,21]. Empathy erosion has been linked to a stressful clinical environment, persistent time pressure, exposure to unemphatic role models, the prioritization of theoretical knowledge over interpersonal skills, and insufficient encouragement of empathic behavior. In this context, students have been shown to adopt coping strategies—psychological adaptations necessitated by adjustment to these environmental demands—such as cynicism, desensitization, and emotional distancing to manage training-related stress, which may contribute to reduced empathic engagement [22,23].

The use of the JSE-HPS allowed for standardized assessment of empathy and comparison with existing literature in dental education. The Cronbach’s alpha coefficient obtained for the Lithuanian version of the JSE-HPS (α = 0.808) falls within the range reported in prior studies (0.77–0.88), supporting the reliability and cross-cultural applicability of the instrument [24,25,26,27,28]. Although the adapted version of the JSE-HPS confirmed the expected three-factor structure, some items exhibited factor loadings that differed from their original theoretical assignments [29]. PT dimension accounted for the largest proportion of items (12 items), whereas fewer items loaded on CC (3 items) and SPS (3 items). In addition, two items demonstrated cross-loadings on both PT and CC, suggesting conceptual overlap between cognitive and affective components of empathy in our sample. Such variations in item–factor associations have been reported in previous cross-cultural adaptations of the JSE-HPS and may reflect contextual or cultural differences in how empathic constructs are interpreted rather than deficiencies in the instrument [26,30,31,32].

The mean total empathy score observed in this sample was 106.07 ± 12.55. Although no standardized cut-off scores have been established for the JSE-HPS, the overall empathy level among Lithuanian dental students appears comparatively high. Specifically, mean empathy scores were higher than those reported among dental students in Malaysia (84.11 ± 9.80) [33], Saudi Arabia (84.84 ± 11.28) [34], and Nigeria (104.01 ± 19.64) [35], but lower than scores reported in Argentina (108.2 ± 5.0) [36], the United States (112 ± 11) [37], and Thailand (114.30 ± 13.06) [38]. The study sample was predominantly female, and previous research suggests that female students tend to report higher levels of empathy than their male counterparts [39,40,41]. This gender distribution may influence the generalizability of the findings; however, a similar female predominance has also been reported in most of the studies used for comparison. The expression of empathy in Lithuania has been strongly shaped by historical and cultural factors, including the legacy of the Soviet repressive system, a prolonged period of insecurity and distrust toward institutions, the Catholic tradition emphasizing suffering and endurance, and the rural community model in which help was offered through actions rather than words. Together, these influences have fostered a restrained, largely nonverbal form of empathy. Minelgaitė Snæbjörnsson et al. [42] observed that Lithuania tends to exhibit characteristics of a high-restraint culture. A study including physicians providing reanimatological and surgical treatment determined that the level of empathy among Lithuanian physicians was statistically significantly lower than that reported for medical professionals in other countries. Additionally, it has been observed that empathy scores in Eastern countries tend to be lower compared with those in Western countries [43]. Based on existing sociocultural literature, emotions are often experienced privately, not displayed publicly, and understood as a personal matter rather than something to be openly shared. Empathy is therefore expressed with caution and modesty, avoiding emotional exposure that might be perceived as weakness or intrusion. In healthcare settings, including dentistry, this often translates into empathy conveyed through professional competence rather than emotional dialog. Many Lithuanian patients feel cared for when treatment is effective, precise, and responsibly delivered [44]. Consequently, Lithuanian healthcare professionals may speak less about emotions or emotional distress while striving to ‘do everything right’ from a clinical standpoint.

We found that empathy declines as students advance in their academic training, with fifth-year students exhibiting significantly lower total empathy scores compared with third- and fourth-year students. Several studies in dental education have reported similar patterns. Kaya et al. [45] evaluated 269 third-, fourth-, and fifth-year students and observed a decrease in empathy from the third to the fifth year, although the difference was not statistically significant. Aggarwal et al. [46] found that graduate students exhibited the lowest empathy levels, while first-year students showed the highest. Similarly, Lee and Ihm [47] reported that students with a shorter academic period in dentistry demonstrated more patient-centered attitudes. Arslan and Hazar Bodrumlu [48] observed a statistically significant gradual decline in empathy among students from the third to the fifth year. Collectively, these findings reinforce the notion that empathy tends to diminish as dental students progress through their training.

Analysis of the JSE-HPS subscales in our Lithuanian sample revealed a decline in PT, reflecting the cognitive dimension of empathy, among fifth-year students compared with third- and fourth-year students. CC, representing the affective component of empathy, decreased significantly between the fourth and fifth years, whereas SPS did not vary significantly across academic years. These patterns suggest that the cognitive and affective components of empathy may be differentially susceptible to erosion during the later stages of dental education. SPS may be relatively resistant to such decline, potentially due to consistent exposure to patient-centered communication and clinical interactions throughout training, which reinforces students’ ability to adopt the patient’s perspective. The limited number of items assessing SPS may also reduce its sensitivity to change over time. Given the similar use of the JSE-HPS and comparable academic stages, our findings can be directly compared with the investigation by Prso et al. [49] in Poland and Croatia. In that study, Polish students exhibited a significant increase in PT from the fourth to fifth year, a non-significant increase in CC, and largely unchanged SPS, whereas Croatian students showed a significant decrease in SPS with no substantial changes in PT or CC. According to the authors, the Polish academic program implements training in communication skills and empathy from the first year and continues throughout the entire course of study. These courses are designed to promote a patient-centered approach and consist of both theoretical lectures and patient-oriented clinical exercises. At the LSMU, dental students complete a course entitled ‘Psychology of Communication’, while at VU they attend the course ‘Basics of Professional Communication and Psychosomatics. Psychiatry’. These courses introduce the basic principles of interpersonal communication and provide theoretical knowledge in the psychology of communication. During the sessions, the specific features of communication with individuals of different age groups are explored. The courses also examine psychological and social factors related to communication, including the processes and errors of social perception, emotions, individual personality differences, and intercultural differences. Even though students in both countries receive communication skills training, overall empathy levels and empathy subscale scores differ markedly between the two cohorts.

Total JSE-HPS scores in our study were positively and significantly correlated with all three subscales, indicating that each dimension contributes to the overall construct of empathy. However, the SPS subscale did not show significant correlations with PT or CC, suggesting that the experiential ability to adopt the patient’s perspective may function independently from cognitive and affective components. This pattern is consistent with findings from the Italian version of the JSE-HPS, which also reported no significant correlation between SPS and PT [50]. Conversely, the Greek JSE-HPS study observed a weak positive correlation between CC and SPS, highlighting that the interrelationships among empathy subcomponents may vary across cultural and educational contexts [51]. Collectively, these findings support the multidimensional nature of empathy in dental students and underscore the importance of addressing cognitive, affective, and experiential components as distinct yet complementary targets in educational interventions.

Several limitations of this study should be acknowledged. First, the cross-sectional design precludes causal inference regarding changes in empathy across academic years, and longitudinal studies are needed to assess within-student trajectories. Second, the study relied on self-reported measures using the JSE-HPS, which may be influenced by social desirability or response bias. Third, although the Lithuanian version of the JSE-HPS demonstrated good internal consistency, its test–retest reliability was not evaluated, limiting conclusions about temporal stability. A major strength of this study is that it included students from the only two universities in Lithuania providing dental education, thereby covering nearly the entire national dental student population. Finally, cultural and curricular factors specific to Lithuania may limit the direct generalizability of these findings to other countries. Despite these limitations, this study provides the first systematic assessment of empathy among Lithuanian dental students, offering valuable insights for educational strategies aimed at fostering empathy throughout clinical training.

## 5. Conclusions

This study demonstrates that the Lithuanian version of the JSE–HPS is a reliable and valid instrument for assessing empathy among dental students. A significant decline in empathy was observed during clinical training, with both the cognitive and affective components markedly reduced in fifth-year students, while the patient-centered perspective component was preserved.

## Figures and Tables

**Table 1:** Participants’ characteristics.

Characteristics	N (252)	%
**Gender**		
**Female**	224	88.89
**Male**	28	11.11
**Age (year), M (SD)**	24.04 (3.08)	
**University**		
**LSMU**	198	78.57
**VU**	54	21.43
**Academic year**		
**Third**	85	33.73
**Fourth**	85	33.73
**Fifth**	82	32.54

M—mean, SD—standard deviation.

**Table 2:** Rotated factor loadings and item statistics.

Item	Factor Structure			Item Statistics			
	F1	F2	F3	Min	Max	Mean	SD
#11	**0.672**	0.205	0.111	1	7	5.43	1.43
#14	**0.642**	0.169	0.091	1	7	5.84	1.33
#2	**0.618**	0.164	−0.183	2	7	6.59	0.84
#20	**0.601**	0.458	−0.080	1	7	6.22	1.20
#7	**0.599**	0.038	−0.040	1	7	6.39	1.03
#4	**0.518**	0.168	−0.138	1	7	6.18	1.08
#15	**0.485**	0.334	−0.222	2	7	5.61	1.37
#19	**0.479**	−0.158	0.113	1	7	5.87	1.47
#1	**0.447**	0.110	0.021	1	7	5.96	1.44
#12	**0.438**	0.194	0.017	1	7	5.01	1.52
#5	**0.404**	0.122	−0.192	1	7	5.35	1.36
#8	**0.383**	0.294	0.148	1	7	5.48	1.61
#17	−0.069	**0.769**	0.122	1	7	4.13	1.60
#9	0.150	**0.724**	−0.35	1	7	5.31	1.45
#13	0.278	**0.623**	−0.159	1	7	5.46	1.44
#16	**0.498**	**0.590**	−0.064	1	7	5.67	1.24
#10	**0.409**	**0.503**	0.041	1	7	5.17	1.45
#6	0.130	−0.085	**0.801**	1	7	3.74	1.50
#3	−0.009	−0.022	**0.727**	1	7	4.19	1.23
#18	−0.087	0.060	**0.445**	1	7	2.48	1.40
**Eigenvalue**	5.29	1.62	1.40				
**% of variance**	24.46	8.09	6.98				
**Cronbach’s α**	0.811	0.639	0.630				

F1—PT (Perspective Taking), F2—CC (Compassionate Care), F3—SPS (Standing in the Patient’s Shoes), SD—standard deviation. Note. Bold values represent item-factor loadings.

**Table 3:** Distribution of empathy scores across academic years.

Academic Year	Empathy Scores					
	Minimum	Median	Maximum	Mean	SE	SD
**Third**	69	109	130	107.05	1.30	11.99
**Fourth**	82	109	135	109.36	1.21	11.74
**Fifth**	67	102	126	101.65	1.47	13.31
**Total**	67	108	135	106.07	0.791	12.55
**Kruskal–Wallis test**				*p* < 0.001		

SE—standard error of mean, SD—standard deviation.

**Table 4:** Post hoc pairwise comparisons of empathy scores by academic year.

Comparisons	Sig.	Adj. Sig. ^a^
**Third-fourth**	0.613	1.00
**Third-fifth**	0.012	0.035
**Fourth-fifth**	0.002	0.007

^a^ Significance values have been adjusted by the Bonferroni correction for multiple tests.

**Table 5:** Distribution of empathy subscale scores by academic year.

Academic Year		PT	CC	SPS
**Third**	Mean	56.56	42.65	7.84
	Median	57.00	43.00	8.00
	SE	0.79	0.71	0.24
	SD	7.30	6.50	2.22
**Fourth**	Mean	57.32	43.85	8.20
	Median	58.00	44.00	8.00
	SE	0.79	0.55	0.27
	SD	7.26	5.09	2.47
**Fifth**	Mean	53.06	40.83	7.76
	Median	54.00	41.00	8.00
	SE	0.98	0.67	0.25
	SD	8.87	6.07	2.23
**Kruskal–Wallis test**		*p* = 0.005	*p* = 0.005	*p* = 0.373
**Total**	Mean	55.68	42.46	7.93
	Median	56.00	43.00	8.00
	SE	0.51	0.38	0.15
	SD	8.02	6.02	2.31

PT—Perspective Taking, CC—Compassionate Care, SPS—Standing in the Patient’s Shoes, SE—standard error of mean, SD—standard deviation.

**Table 6:** Post hoc pairwise comparisons of empathy subscales by academic year.

Comparisons	PT		CC	
	Sig.	Adj. Sig. ^a^	Sig.	Adj. Sig.^a^
**Third-fourth**	0.613	1.00	0.294	0.881
**Third-fifth**	0.012	0.035	0.030	0.091
**Fourth-fifth**	0.002	0.007	0.001	0.004

PT—Perspective Taking, CC—Compassionate Ca. ^a^ Significance values have been adjusted by the Bonferroni correction for multiple tests.

**Table 7:** Pearson correlations between JSE–HPS total and subscale scores.

	PT	CC	SPS
**Total JSE-HPS**	0.890 **	0.830 **	0.139 *
**PT**		0.568 **	−0.053
**CC**	0.568 **		−0.008
**SPS**	−0.053	−0.008	

PT—Perspective Taking, CC—Compassionate Care, SPS—Standing in the Patient’s Shoes; * correlation is significant at the 0.01 level; ** correlation is significant at the 0.05 level.

## Data Availability

The data presented in this study are available on request from the corresponding author due to privacy reasons.

## Abbreviations

The following abbreviations are used in this manuscript:

JSE-HPS	Jefferson Scale of Empathy–Health Professions Students
PT	Perspective Taking
CC	Compassionate Care
SPS	Standing in the Patient’s Shoes
LSMU	Lithuanian University of Health Sciences
VU	Vilnius University
SD	Standard deviation
SE	Standard error of the mean
EFA	Exploratory factor analysis
KMO	Kaiser–Meyer–Olkin
